# Expert Perspectives on Enhancing Analytical Methods for Multi-Ingredient Dietary Supplements (MIDS): A Qualitative Study

**DOI:** 10.3390/foods14213598

**Published:** 2025-10-22

**Authors:** Ingyeong Ko, Hae Jin Park, Kwang Suk Ko, Hyunsoo Kim, Jieun Oh

**Affiliations:** 1 Department of Nutritional Science & Food Management, Ewha Womans University, Seoul 03760, Republic of Korea; dlsrudrh9954@ewha.ac.kr (I.K.); jin96@ewhain.net (H.J.P.); kko@ewha.ac.kr (K.S.K.); 2 Department of Convergent Bioscience and Informatics, Chungnam National University, 99 Daehak-ro, Yuseong-gu, Daejeon 34134, Republic of Korea; kimlab@cnu.ac.kr; 3 College of Science & Industry Convergence, Ewha Womans University, Seoul 03760, Republic of Korea

**Keywords:** multi-ingredient dietary supplements (MIDS), complex raw materials, formulation, analytical methods, ingredient interactions, quality control

## Abstract

The increasing demand for multi-ingredient dietary supplements (MIDS), driven by diverse consumer health needs, has introduced analytical challenges in product testing and quality control. These challenges stem from complex ingredient interactions, formulation variability, and the diverse physicochemical properties of the individual components. To examine these issues and explore practical solutions, this study employed semi-structured focus group interviews with 33 industry professionals and 10 analytical experts from academic and industry. Professionals reported major obstacles including the degradation or loss of trace components, interferences among ingredients, analytical difficulties with specific dosage forms, and the lack of standardized testing protocols. To mitigate these challenges, professionals reported implementing various combination strategies including substituting problematic raw materials and modifying analytical instruments and pretreatment procedures, in order to improve test reproducibility. These measures were developed internally and varied significantly across companies, reflecting the absence of a unified analytical framework for MIDS testing. Building on these insights, the analytical experts proposed systematic improvements including developing matrix-specific pretreatment protocols and optimized extraction strategies as well as regulatory harmonization to enhance analytical reliability and reproducibility. These findings provide critical insights into current field practices and inform the development of standardized methodologies for the analysis and quality assurance of MIDS.

## 1. Introduction

The growing emphasis on health and the adoption of preventive lifestyles have driven a marked increase in the global demand for dietary supplements (DSs). This trend intensified following the declaration of the COVID-19 pandemic in March 2020, as heightened public interest in immune support and disease prevention led to a surge in the consumption of vitamin- and mineral-based DSs [1].

According to a 2024 report by Statista, the global DS market is valued at approximately USD 194.56 billion and is projected to grow to USD 211.68 billion by 2025 and USD 415.63 billion by 2033, reflecting a compound annual growth rate (CAGR) of 8.80%. Regionally, the North American market is expected to grow at a CAGR of 9.55%, while the Asia-Pacific region is projected to expand at 8.9%.

Among the most dynamic segments in this expanding market is multi-ingredient dietary supplements (MIDSs), which combine various functional components into a single product to meet the consumer demand for convenience and comprehensive health support [2]. MIDSs have become one of the most widely consumed supplement types, with over one-third of adults in the United States and Europe reportedly using them daily [3]. To meet diverse nutritional needs, manufacturers have introduced MIDSs in a variety of combinations and dosage forms including tablets, soft capsules, chewables, and jellies [4].

However, the combination of multiple active ingredients within a single formulation presents significant challenges. These include inter-ingredient interactions, stability issues, and analytical complications. For instance, liquid multivitamins may require different storage conditions for individual vitamins, complicating stability management for the final product [5]. In another study, discrepancies between the labeled and actual CoQ10 content were observed in commercial products. While rCoQ10 exhibited relative stability, oCoQ10 degraded during storage. Additionally, vitamin C was observed to impede the oxidation of oCoQ10; however, its safety profile was found to be contingent upon the storage conditions [6].

Despite the complexity of MIDS formulations, most existing research has concentrated on ingredient safety [7,8,9], physiological interactions [10,11,12], or bioavailability [13,14,15]. However, relatively few studies have addressed the practical challenges encountered during the formulation, manufacturing, and analytical testing of MIDSs [16].

To address this gap and gain deeper insights into the practical challenges faced in industrial settings, it is essential to explore the contextual factors influencing these challenges. Focus group interviews (FGIs) offer a robust qualitative method for capturing diverse field-based experiences and expert insights [17,18].

Accordingly, this study aimed to investigate the practical challenges associated with the manufacturing, formulation, and quality control of MIDSs. Through FGIs with industry and analytical experts, the study sought to identify field-based issues, explore potential solutions, and generate foundational data to improve testing methods tailored to the complexity of functional composite ingredients and diverse formulations, thereby enhancing their practical applicability.

## 2. Materials and Methods

### 2.1. Study Design

This study was conducted in two phases of FGIs to systematically identify the challenges encountered in the production and analysis of MIDSs and explore practical solutions. The terminology used throughout this paper follows the Standards and Specifications for Health Functional Foods published by the Korean Ministry of Food and Drug Safety. These standards are hereafter referred to as the MFDS standards. In accordance with the Ministry of Food and Drug Safety [19], MIDSs are defined as products containing two or more functional ingredients.

**Figure 1:**
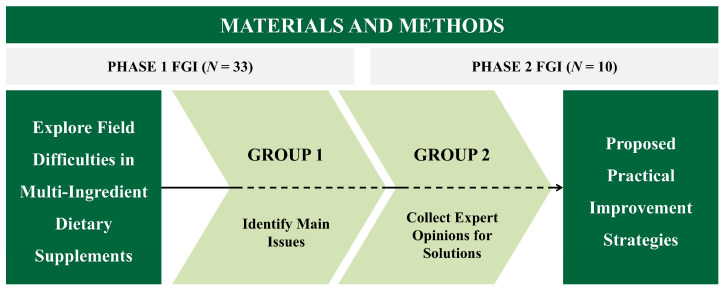
Materials and methods.

In Phase 1, FGIs were conducted with professionals from the manufacturing, analytical, and quality control sectors to explore field-level challenges. In Phase 2, FGIs were conducted with analytical experts from both academia and industry to develop solutions to the problems identified in Phase 1 (Figure 1).

This study received ethical approval from the Institutional Review Board of Ewha Womans University (Approval No. ewha-202405-0035-02), and all participants provided informed consent.

### 2.2. Participants

Participants were selected through purposive sampling, a qualitative method chosen to ensure the inclusion of professionals with relevant expertise across raw material manufacturing, distribution, and analysis within the MIDS industry as well as academic and industrial experts in quality and analytics. A detailed recruitment notice outlined the study’s objectives and eligibility criteria. Participation was voluntary, and written informed consent was obtained prior to data collection.

A total of 43 people participated in the study. Industry professionals participated in the first-stage focus group interview (FGI), and academic and industry experts participated in the second stage FGI. This sample size was used to thoroughly explore the issues and solutions in the field, reflecting the participants’ diverse professional experiences and expertise. During data collection, repeated observation of the participants’ statements revealed that new information did not significantly alter the previously developed codes, leading to data saturation. Consequently, data collection was terminated.

### 2.3. Procedure

The FGIs were conducted between 3 June 2024 and 20 March 2025. Each session lasted approximately 60 min and used a semi-structured questionnaire (Table 1 and Table 2). The questionnaire was designed to deeply explore the on-site experiences of MIDS industry professionals and experts. It drew on prior research reporting analytical challenges in the field [20,21], aligning with the study’s objectives. It reflected issues repeatedly reported in previous studies such as interactions between raw materials and ingredients, analytical limitations of different formulations, and difficulties in applying testing methods. The interviews were organized into two distinct phases, with questions tailored to the roles and expertise of the respective participant groups.

Phase 1 included FGIs with professionals involved in raw material manufacturing, distribution, and analysis. The discussions focused on four key areas: (i) raw material and ingredient-related challenges, (ii) formulation-related challenges, (iii) analytical method-related challenges, and (iv) solutions currently implemented in the field.

Phase 2 included FGIs with analytical and quality control experts from academia and industry. This phase focused specifically on generating solutions to the challenges identified in Phase 1.

All FGIs were audio-recorded with prior consent and transcribed verbatim for analysis. To ensure confidentiality, audio recordings were permanently deleted following transcription. The interview transcripts were analyzed using thematic analysis.

### 2.4. Analysis

The research team transcribed all interview recordings using ClovaNote and reviewed the accuracy of the transcripts. Then, the transcripts were anonymized. The transcripts were sent to the participants via email, and they were asked to respond within one week with corrections to any inaccuracies or misrepresentations. The final transcript was analyzed using NVivo 15.2.1 (Lumivero Pty Ltd., Burlington, MA, USA, 2024) according to Braun and Clarke’s (2006) [22] reflective thematic analysis procedure. Two researchers conducted independent coding, and any discrepancies were resolved through consensus.

**Figure 2:**
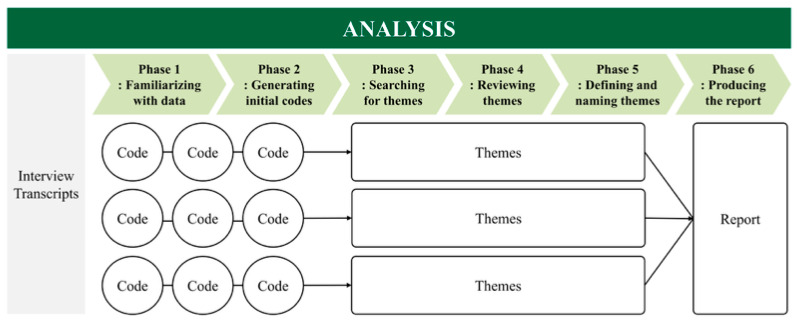
Thematic analysis process applied in this study.

Thematic analysis was employed to identify, interpret, and organize recurring patterns within the qualitative data [23]. This study used a six-step procedure to structure the complex issues derived from interviews with numerous MIDS industry practitioners and quality and analysis experts from academia and industry. The procedure consisted of familiarizing with the data, generating initial codes, searching for themes, reviewing themes, defining and naming themes, and producing the report [22] (Figure 2).

In the first-stage of reflective thematic analysis, familiarizing with the data, the transcribed text was read repeatedly, and initial notes were taken to become familiar with the data and set the direction of the analysis. In the second stage, generating initial codes, meaningful units were identified in the text and coded. Initial coding was conducted while maintaining the flow and context of the data. The focus was on recurring characteristic statements and ensuring that key elements related to the research questions were not omitted. In the third stage, searching for themes, the generated codes were grouped into higher-level concepts to derive potential themes. In this process, similar or related codes were integrated and categorized. Fourth, during the reviewing themes stage, the themes were examined to ensure consistency with the data’s overall context. Specifically, the analytical framework’s validity was confirmed by first ensuring that each theme matched the coded data, and then checking its consistency with the entire dataset. Fifth, during the defining and naming themes stage, the specific characteristics and meanings of each theme were refined, and names were assigned to clarify the relationships between themes. Sixth, during the producing the report stage, representative quotations and examples were selected for each theme, and the results were described. Finally, the derived themes were discussed in an academic context in relation to the research questions and previous studies.

## 3. Results

### 3.1. Participant Characteristics

The demographics of study participants are summarized in Table 3. A total of 33 professionals participated in the Phase 1 FGIs, with the majority working in analytical and quality control roles (20 participants), followed by research and development (10 participants), and materials research (3 participants). The average work experience in this group was 15 years.

A total of ten experts participated in the Phase 2 FGIs, with the majority working in analytical roles (seven participants) and the remaining in DS development and product planning (three participants). Their average professional experience exceeded 17 years.

Thematic analysis of the interview transcripts yielded 4 main themes, 7 sub-themes, and 16 codes (Table 4). A detailed explanation of each theme, along with supporting participant quotations, is provided in Table S1.

### 3.2. Phase 1 FGI Analysis Results

#### 3.2.1. Raw Materials and Ingredients

- Difficulties in the analysis of raw materials and ingredients

Professionals reported significant challenges in the analysis of raw materials and ingredients, particularly due to low detection sensitivity and variability in processing conditions. Trace elements such as vitamins D, K, and B12 exhibited inconsistent results despite using the same protocols, often due to factors such as the pretreatment method, analyst proficiency, and instrument calibration. This lack of method validation impairs analytical reproducibility.

“Trace elements can get buried, and it is very difficult to actually see them.”

(Professional #1)

“Trace ingredients like vitamin D and vitamin K are sometimes all detected when the content is validated, but in some situations, only half of them are detected.”

(Professional #7)

Notably, certain ingredients, such as pantothenic acid, naturally degrade over time and may fall below the regulatory thresholds before expiration. To mitigate this, the Health Functional Food Code permits content overdosing up to 180% [19].

“If the overdose is not applied, the content falls below the standard within the expiration date, and stability is compromised.”

(Professional #1)

Analytical challenges were also reported for naturally derived ingredients, with biases in results arising from differences between these ingredients and their synthetic reference materials [24]. For example, in Acerola fruit extracts, even when analyzing the same vitamin C component, peak time differences compared with their synthetic counterparts led to analytical errors. Similarly, in the analysis of Rosavin from Rhodiola rosea L. extracts, quantification was complicated by overlap with isomers and impurities in the testing process.

“Vitamin C in Acerola fruit extracts has a different peak time than synthetics, which can cause differences in analysis.”

(Professional #4)

“I could see that the test for Rhodiola rosea L. extracts tended to have some impurities in it.”

(Professional #16)

Moreover, pH and temperature sensitivity influenced the analytical accuracy. Folic acid, for instance, displayed peak mismatches when standard and sample solutions had differing pH values [25]. Ginsenosides were similarly affected, showing structural changes under acidic or thermal conditions [26].

“Folic acid, for example, I think it is a little bit affected by pH.”

(Professional #14)

“Ginsenosides themselves are affected by acid. It is affected a little bit by temperature and pH, so Rg1 becomes Rg3, and there’s damage like that.”

(Professional #15)

- Analysis and interaction issues in composite ingredients

In multi-ingredient formulations, ingredient interactions frequently compromised analytical reliability. These effects included overestimation, under-detection, and instability of measured values.

Over-quantification was common in ingredients like bilberry and cranberry extracts, where structurally similar compounds were simultaneously detected due to overlapping absorbance wavelengths. Similar issues occurred with carotenoids such as lutein, which co-eluted with zeaxanthin and astaxanthin.

“When analyzing cranberry fruit extracts, similar anthocyanosides are sometimes detected together in the absorbance, resulting in a high content.”.

(Professional #2)

“When analyzing lutein, other carotenoids such as astaxanthin, zeaxanthin, and beta-carotene can be detected together, which can skew the results.”.

(Professional #10)

On the other hand, under-quantification was also reported. Vitamin B12 content significantly declined or became undetectable when mixed with various compounds including R. rosea L. extracts, vitamin C, copper sulfate, or milk thistle extracts [27,28]. Similar issues were found with vitamin D when combined with rice bran extract. Catechins were also reduced by more than half when mixed with amino acids, despite remaining stable when tested alone [29].

“It is a method that is in the Health Functional Food Code, but when catechins and amino acids were mixed, the process was done according to the process, but when the raw materials were tested alone, the result was 100, but when amino acids were mixed, it did not even come out half.”

(Professional #15)

“I once used organic raw materials such as vitamin D and rice bran extract as secondary raw materials, but when the two were mixed together, the vitamin D content did not come out.”

(Professional #19)

Additionally, trace components such as biotin and vitamin D were particularly vulnerable to degradation or chemical modification when combined with excipients, likely due to sensitivity to pH, temperature, or adulterants [30].

Variability in test results based on formulation and test conditions was a recurring concern. Professionals highlighted that factors such as the choice of method, pretreatment, and formulation composition often interacted to affect analytical precision [31]. For instance, vitamin B2 demonstrated instability in formulations with high magnesium content.

“If you mix vitamin B2 in a high magnesium product, the value of vitamin B2 is a little bit unstable.”

(Professional #14)

Ingredient interference further complicated the analysis. In propolis extracts, excipients such as Saururus chinensis influenced flavonoid absorbance, skewing results. Natural excipients in vitamin C products (e.g., lemon extract powder) also led to unanticipated component detection or measurement errors.

“The flavonoids in propolis extracts are highly interfered with by excipients, and the variations are quite high.”

(Professional #2)

“There was also interference between the individual recognizable forms of Saururus chinensis extract and propolis extracts.”

(Professional #19)

“I put 100% inputs into the vitamin C product, but there was interference because due to vitamin C in the excipients… ”

(Professional #3)

“Saw palmetto fruit extracts have a lot of assay variation and does not give the right assay.”

(Professional #11)

“When you mix natural ingredients with natural ingredients, it becomes unstable.”

(Professional #10)

#### 3.2.2. Formulation

With the diversification of MIDS product forms, the physical characteristics of each formulation have introduced increasingly complex analytical constraints. Industry professionals reported that existing test methods often fall short, particularly for newly introduced formulations, which may not be appropriately analyzed using current procedures.

- Analytical challenges arising from formulation diversity

Jelly formulations were identified as particularly difficult to analyze. Since these formats were developed after most test methods were established, existing protocols often fail to accommodate the unique properties of jellies. Analytical challenges include issues related to viscosity, incomplete dissolution, and variability caused by cutting methods. In some cases, the jelly would stick during grinding, further disrupting accuracy [32]. To address this, various experimental approaches were attempted such as preheating for complete dissolution and adjusting sample volumes. However, due to the lack of suitable test methods, some companies paused production or switched to alternative formulations.

“There are some things that the existing formulations cannot cover because the formulations were created first and the jelly formulation was added on.”

(Professional #2)

“Some of the formulations we’ve had a little trouble with are jelly.”

(Professional #14)

“With jelly, you do not melt it all the way through, and then you end up cutting it up, and now you have to analyze it, and depending on how much you cut it up and what size you cut it into, you’re going to get different amounts.”

(Professional #15)

Soft capsules also pose analytical difficulties, particularly in separating the outer shell from the active contents. This can lead to the loss of analytes or degradation during pretreatment. For example, soft capsules containing water-soluble vitamins require water-based pretreatment solvents, but these often fail to fully dissolve the contents due to the capsule’s properties.

“With water-soluble vitamins in soft dosage forms, there are challenges with water-based pretreatments that do not sufficiently dissolve the ingredients.”

(Professional #9)

Difficulties in analysis due to migration into the film within the soft capsule were also mentioned [33], leading to the need for MIDSs, like pharmaceuticals, to expand soft capsule testing for a wider range of ingredients, not just water-soluble vitamin content.

“It is not that the ingredient is not actually present, but that it has been transferred to the film. To prove this, you have to repeat experiments for each formulation, but it is difficult to continue this in reality.”.

(Professional #6)

It was mentioned that solid formulations are prone to deviation in analytical values because they are not completely water-soluble due to interference factors. Among solid formulations, granular formulations are affected by the inclusion of acid in the composition, which changes the activity of water-soluble vitamins. This may result in poor separation and a phenomenon in which the detection peak spreads.

“The addition of acid in multivitamin granules or wet formulations can cause dissipation problems, depending on the activity of the water-soluble vitamins.”.

(Professional #4)

Professionals also emphasized that current standards, such as those in the Standards and Specifications for Health Functional Foods, primarily addressed older or conventional dosage forms. As a result, newly introduced formulations lacked the appropriate analytical guidelines or pretreatment instructions. A recurring recommendation was the need for formulation-specific test methods, particularly standard operating procedures (SOPs) tailored to each formulation type.

“I also think now that test methods need to be formulation specific.”

(Professional #13)

#### 3.2.3. Testing Methods

Challenges concerning test methods were raised in two main areas: the absence or limited applicability of existing methods, and variability caused by differences in chromatography columns used by the manufacturers. Both factors were identified as significant barriers to achieving reliable and consistent analytical results, impacting product quality assessment.

- Limitations of current testing methods

Professionals reported that many MIDSs lacked suitable test methods, or that current methods had limited practical use. For instance, standardized methods for ingredients like glutathione and Saw palmetto fruit extracts are either absent from the Standards and Specifications for Health Functional Foods or fail to produce valid results. The acetone-insoluble phospholipid test for krill oil, as specified in the standards, was found to be unreliable, showing discrepancies exceeding by 30% from the actual content.

“There is no separate method for glutathione, so it is difficult. Also, all phospholipids are supposed to be checked by the acetone-insoluble method, but when the phospholipids of krill oil are checked by the acetone-insoluble method, the data difference is about 30%, so it cannot be verified.”

(Professional #1)

The lack of analytical standards for newer formulations was also highlighted. For example, stick jelly formulations require melting at elevated temperatures (40–50 °C), but specific testing guidelines for this are absent, complicating their practical analysis. Additionally, discrepancies exist between test methods listed in the Food Standards and Specifications and those in the Standards and Specifications for Health Functional Foods. For instance, biotin is more easily detected using food methods, but it is challenging to detect using DS methods.

“When using the food method and the dietary supplements method, biotin was better analyzed in the food method and so on.”

(Professional #14)

“The stick jelly melts at 40 to 50 degrees, but that is not in the instructions.”

(Professional #29)

Another concern was the lack of clearly defined pretreatment protocols in the standards, leading to varied sample preparation approaches by analysts analyzing the same ingredient. This variability undermines the reliability and objectivity of testing.

“I think it would be helpful to have a little more information in the manual about why they used these solvents in the instructions.”

(Professional #17)

A clear specification of the test conditions and pretreatment procedures in future method revisions was strongly recommended.

- Inter-manufacturer differences in chromatography column performance

A key challenge identified was the variability in the performance of chromatography columns used by different manufacturers. Despite adhering to the same specifications, columns may differ in packing technology and quality, leading to inconsistencies in retention time, peak shape, and resolution, which are factors that directly influence the accuracy of content determination [34].

“Technical differences between manufacturers can lead to differences in separation, sharpness, etc., and the retention time itself can vary.”

(Professional #2)

These inconsistencies have been observed even when the column specifications outlined in the Standards and Specifications for Health Functional Foods are strictly followed. This has led some analysts to advocate for using columns from a specific brand or manufacturer as a standard reference. Notably, even filters or cartridges made from the same material have been reported to vary by more than 20% between suppliers. Such variability poses a significant risk to reproducibility, quantitative accuracy, and the overall objectivity of analytical standards.

“It is important to stick to the column that was used as a specific reference standard.”

(Professional #7)

### 3.3. Phase 2 FGI Analysis Results

#### Expert-Recommended Analytical Solutions

- Tailored pretreatment and extraction strategies

To address analytical challenges related to complex raw materials and formulations, experts recommended tailored pretreatment techniques, the adjustment of separation conditions, enzyme utilization, and the standardization of sample preparation based on component characteristics. Practical solutions were proposed to tackle issues such as interactions, stability degradation, and quantitative inconsistencies including adapting the pretreatment to standards, optimizing enzyme-based extraction, adjusting packing materials, and modifying the mobile phase composition.

Several experts suggested strategies to mitigate component degradation caused by interactions in MIDSs, focusing on key ingredients like vitamin B12, vitamin K2, vitamin B9 (folic acid), and R. rosea L. extracts. For vitamin B12, periodic enzymatic hydrolysis (e.g., pepsin treatment) improves extraction by freeing the component from complex matrices [35]. Vitamin K2, which is prone to analytical variability due to its fat-soluble nature, benefits from lipase enzyme treatment during pretreatment [36]. For folic acid, pretreatment with 0.1 N NaOH followed by elution with 10 mM phosphate buffer aligns the sample with the standards, addressing inconsistencies [19]. Additionally, the separation of rosavin isomers in Rhodiola extracts can be improved by reducing the filler size or using buffered mobile phases.

“For vitamin B12 analysis, depending on the matrix nature of the sample, an additional enzymatic hydrolysis treatment is required to free the component from the matrix so that it can be extracted using an enzymatic treatment method such as pepsin… Vitamin K requires different pretreatment or extraction methods, such as lipase enzyme treatment to break down fats present in the sample.”

(Expert #3)

“The sample pretreatment is the same as for the standard, a certain amount of sample is taken, dissolved with 0.1 N NaOH, and eluted with 10 mM phosphate buffer for analysis… The separation of rosavin isomers and rosavin in Rhodiola rosea extracts is considered possible by reducing the size of the column filler or by using buffer as the mobile phase.”

(Expert #6)

For formulation-specific challenges, the experts proposed targeted pretreatment methods. Jelly formulations may require methods for cutting into small pieces, controlling the amount of sample, and pretreatment methods for completely melting the jelly were suggested. Protein gels benefit from dispersion in water or pH buffer, followed by acetone extraction. Soft capsules can be pretreated by separating the shells using dichloromethane or chloroform. Solid dosage forms are best homogenized with membrane bowls before analysis, and coated tablets require coating removal to avoid interference.

“In the case of jellies, we are now experimenting with chopping them up as much as possible and controlling the sample volume. In addition, you can try freezing the jelly completely in liquid nitrogen.”

(Expert #10)

“You can use dichloromethane or chloroform to separate them and then do the experiment.”

(Expert #7)

- Regulatory improvements and method development

To improve test methods, experts recommended selecting representative matrices for each product type and tailoring the analysis conditions accordingly. They highlighted the need to consider both universal and advanced, possibly costly, analytical equipment.

“The test method generally recommends using general-purpose and universal equipment, but it would be good to consider expensive equipment as well, because there may be expensive equipment or equipment that not everyone can afford.”

(Expert #5)

Regarding the test methods in the Standards and Specifications for Health Functional Foods, the experts suggested adopting internationally recognized methods or allowing for the parallel application of multiple standards to improve flexibility. However, they also warned of potential inconsistencies between methods and stressed the need for clear interpretation guidelines and management systems.

“It is good to have multiple analytical methods, but as the number of analytical methods increases, the analyst needs to be responsible for whether it is appropriate to use the method.”

(Expert #7)

To address regulatory and technical limitations in the application of test methods, the experts emphasized the need for more flexible management systems. Since uniform standards are currently applied to all MIDSs despite diverse formulations, they proposed developing representative standards by product type, introducing overseas methods, customizing analyzes based on ingredients, and ensuring responsible oversight of test applications.

“For the products developed so far, it is necessary to divide them into several types, select a representative matrix, and develop appropriate analytical methods.”

(Expert #4)

“It will be difficult to apply all methods from the Health Functional Food Code to all matrices, but this part needs to be developed through continuous research as in other countries.”

(Expert #3)

### 3.4. Integrated Findings from Multi-Phase FGIs

The first phase of the FGI involved interviewing MIDS industry professionals about the analytical challenges and test method issues. Based on these findings, the second phase engaged quality and analytical experts from academia and industry to propose solutions to the key challenges identified. The results are summarized in Table 5.

## 4. Discussion

This study conducted FGIs with 33 professionals from the MIDS industry and 10 quality and analytical experts from academia and industry to identify key analytical challenges and propose practical solutions.

Raw material interference emerged as a major source of analytical bias. Vitamin D, in particular, was identified as difficult to analyze reliably due to variations in the measured values depending on the pretreatment conditions, pH, temperature, and light exposure [21]. These issues were further compounded in multi-ingredient formulations. In some cases, vitamin D was either undetectable or significantly diminished when combined with other materials such as rice bran extract. Similar interferences were observed for vitamin B12 when formulated with excipients like R. rosea L. extracts, copper sulfate, vitamin C, and milk thistle extracts. These findings suggest that ingredient interactions can have a direct impact on the assay outcomes.

The current literature and regulatory test standards have predominantly focused on the analysis of single ingredients, with minimal consideration of the challenges posed by complex multi-ingredient formulations. For example, Liao et al. (2022) focused on individual ingredient interactions but did not fully explore analytical interferences or quantification errors arising in actual commercial MIDSs [37]. In this study, consistent under-detection of trace nutrients such as vitamin B12, vitamin D, catechins, and biotin was attributed not only to ingredient interactions, but also to limitations in the sensitivity of existing analytical methods. These are not merely technical anomalies—these have direct implications for assessing product functionality and safety, highlighting the urgent need for updated and robust test protocols.

Furthermore, the current testing methods specified in the Standards and Specifications for Health Functional Foods were found to be primarily suited to traditional dosage forms like tablets and capsules and do not adequately address the pretreatment and analytical conditions required for newer formulations such as jellies, soft capsules, and liquids. Consequently, analytical practitioners have often made unsystematic adjustments to sample preparation methods based on formulation type, leading to inconsistencies in the test results. This indicates a clear need to develop formulation-specific analytical guidelines and standardized pretreatment procedures.

The present study also confirmed that variability in ingredient content throughout the product distribution phase remains a significant concern. As previously reported by Djaoudene et al. (2023), and further supported by the current findings, some manufacturers tend to formulate their products with higher initial concentrations of certain ingredients to compensate for anticipated degradation during distribution [2]. While this approach is intended to maintain efficacy until the end of the shelf life, it risks unintentional overconsumption. Andrews et al. (2017) reported similar findings in the U.S., where the discrepancy between the labeled and actual contents of nutrients like selenium and iodine exceeded 25% [38]. Such inconsistencies can hinder accurate dietary intake assessments and reinforce the importance of rigorous, standardized content verification practices.

Methodological weaknesses within the current regulatory framework were also identified. Many listed methods are based on single-ingredient models and fail to account for the interactions that occur in composite formulations. Additionally, inconsistencies across laboratories, due to differences in column performance, pretreatment methods, and incomplete method coverage, pose significant barriers to harmonized quality control. Patel (2020) noted that such issues go beyond mere analytical sensitivity and point to broader systemic problems [39]. To address this, it is essential to develop matrix-based test designs and implement standardized operating procedures across institutions, as emphasized by Williams et al. (2023) [40].

In light of these findings, it is evident that the current testing approaches centered around single-ingredient analysis are insufficient for addressing the complexity of modern MIDSs. A shift is needed toward analytical frameworks that account for multi-ingredient interactions and emphasize stability-based quantification. This includes developing validation schemes that reflect operational variability, creating formulation-specific guidelines, and employing tools that can predict interference risks at the design stage. Coskun et al. (2021) emphasized the importance of method validation that considers the actual product composition and characterization to increase study reproducibility and comparability, an idea supported by this study’s findings [41].

Additionally, as the production and consumption of MIDSs continue to expand globally, the need for internationally harmonized testing standards becomes increasingly critical. Durazzo et al. (2022) argue for the establishment of globally aligned analytical frameworks to support interoperability and regulatory convergence [42]. These efforts will ultimately enhance the credibility and safety of MIDSs and support more effective collaboration between industry and regulatory bodies.

A major strength of this study is that it moves beyond theoretical analysis by directly incorporating the experiences and insights of field professionals and subject matter experts. This provides a grounded understanding of practical issues that are often missed in lab-based studies. The qualitative approach, in particular, allowed for the collection of rich and specific data regarding on-the-ground challenges and current workarounds in the industry, as highlighted by O’Mahony et al. (2023) [43]. These findings lay a strong foundation for future research aimed at developing comprehensive test method guidelines. Going forward, it will be important to build on this qualitative base by incorporating quantitative stability studies that span diverse formulations and manufacturing contexts. The establishment of a robust test method validation system will be key to improving the reliability of MIDS quality assessments.

Nevertheless, this study has several limitations. First, the small number of participants may limit the generalizability of the results. However, qualitative research focuses less on statistical generalization and more on deriving reliable insights into the research topic through the in-depth experiences of expert participants after reaching a certain level of data saturation [44]. The participants in this study comprised industry and academic experts with an average of 16 years of experience. Their practical experiences enabled a concrete exploration of the problems encountered in the field and their solutions. Future research should validate and expand the qualitative insights gained from this study through larger-scale quantitative investigations.

Second, due to the nature of focus group interviews (FGIs), the opinions of one participant may influence those of others, which can be advantageous for deriving collective insights but may also limit the convergence of individual opinions. Third, while this study systematically analyzed issues raised by field practitioners and solutions proposed by experts, this role distinction may have insufficiently reflected cases where problems were relatively less experienced. Some companies may have minimized analytical difficulties through niche technologies, proprietary pretreatment processes, or robust internal quality control systems [45,46]. In-depth analysis of such exceptional cases could provide crucial clues for identifying success factors in test method improvement. Therefore, future research should include these positive cases and corporate-level response strategies to comprehensively understand not only the barriers, but also the enablers within the MIDS industry.

Furthermore, this study was conducted primarily with participants in Korea, limiting its international generalizability. However, issues such as interactions between ingredients, formulation-specific test failures, and discrepancies between the labeled and measured values have also been consistently reported in existing international studies [47,48,49], supporting that the findings may offer implications beyond Korea to the global MIDS industry. Specifically, the USP has focused for over 200 years on establishing quality standards for pharmaceuticals, dietary supplements, and foods to enhance the safety and reliability of the global supply chain. EFSA, established in 2022 as an independent scientific advisory body within the EU, has been tasked with assessing risks across the entire food chain and providing the scientific basis for food-related legislation and regulations [50,51]. The direction of these international organizations aligns with the needs identified in this study: ensuring ingredient safety, establishing pretreatment guidelines for each formulation type, and the necessity for international standardization. This supports the argument that improving MIDS testing methods and addressing formulation-specific analytical limitations should be discussed at a global level, and not confined solely to Korea. Therefore, future research requires comparative analyses encompassing diverse countries, companies, and product groups. It should strengthen the linkage between the MFDS regulatory framework and international standards such as USP and EFSA to ensure the international standardization and interoperability of test methods.

Consequently, this study provides foundational data by structuring and analyzing practical problems repeatedly raised by the MIDS industry and academia and by seeking corresponding solutions. It holds particular significance for presenting a direction for practical test method improvements in the MIDS field.

## 5. Conclusions

This study identified key challenges in the quality control and analysis of MIDS through FGIs with industry professionals and expert consultations. A central issue was the interference caused by co-ingredients, which frequently led to reduced detection or the degradation of target compounds such as vitamin B12, vitamin D, and catechins. These interactions were especially problematic for trace and natural ingredients, where environmental conditions and formulation complexity significantly impacted the analytical outcomes.

Certain formulations, such as jellies, soft capsules, and protein gels, posed additional barriers to accurate analysis due to their physical properties, which compromised ingredient stability and extraction efficiency. These issues were further compounded by limitations in the existing testing methods that were primarily designed for single ingredients or conventional forms like tablets and capsules. Inconsistencies in results due to formulation effects, lack of standardized pretreatment protocols, and variability across chromatography columns all underscore the urgent need for more robust analytical frameworks.

Expert insights also highlighted the necessity of tailoring test methods to formulation characteristics and developing standardized procedures to minimize inter-laboratory variation. Improvements such as enzyme-assisted hydrolysis, buffer system adjustments, and formulation-specific pretreatment strategies were identified as practical measures to enhance accuracy and consistency.

Ultimately, this study emphasizes the importance of understanding ingredient interactions and matrix effects in MIDSs to ensure reliable quality assessments. Developing validated, formulation-specific testing protocols and harmonized analytical guidelines will be critical to strengthening quality control across the industry.

Future research should expand the scope of case studies to include a broader range of companies and products. Additionally, increased collaboration between industry stakeholders and regulatory bodies is essential for translating practical insights into regulatory frameworks. These efforts will support the delivery of safe, effective MIDSs to consumers and enhance the long-term sustainability and competitiveness of the DS sector.

## Figures and Tables

**Table 1:** Phase 1: MIDS ^1^ industry workers.

Topic	Questions
Participant demographics	What is your main task? How much experience do you have?
Raw material and ingredient	What materials and raw materials do you frequently handle or analyze at your workplace? Have you ever felt uncomfortable with the raw materials and ingredients you frequently handle or analyze in your workplace? In what areas have you felt uncomfortable? Have you ever experienced difficulties in component analysis due to interference between functional and auxiliary raw materials? (e.g., raw materials A and B are not mixed) How do you perform analysis for raw materials that are difficult to analyze? Do you have difficulties in component analysis depending on the number of functional raw materials? (Is component analysis more difficult when more than two are mixed?)
Formulation	Are there any cases where you have difficulty analyzing ingredients when making a product in a particular formulation? Are there any formulations where it is particularly difficult to analyze the ingredients? (e.g., soft capsules, jelly formulations, tablets, etc.)
Testing methods	Could you share some of the failures associated with test method development? (trial and error) In the case of columns, are there differences between manufacturers when equivalent columns are available? Product groups currently on the market contain a variety of formulations and functional raw materials. Have there not been cases where they were difficult to analyze using standard testing methods? What do you think about splitting the standard test method by formulation? What additional things do you think are needed to manage the test method according to the material being analyzed?
Other	Other opinions

^1^ MIDS = multi-ingredient dietary supplement.

**Table 2:** Phase 2: Academic and industry experts.

Topic	Questions
Participant demographics	What is your main task? How much experience do you have?
Expert-recommended analytical solutions	Are you aware of the main difficulties in analyzing the components of functional composite raw materials? Have you experienced analytical difficulties due to interactions between composite materials? (e.g., vitamin B12 + copper sulfate, vitamin B12 + milk thistle extracts, vitamin B12 + Rhodiola rosea L. extracts, etc.) Have you experienced analytical difficulties due to interactions between raw materials and excipients? (e.g., saw palmetto fruit extracts + Ginkgo leaf extracts, natural product + natural product, etc.) Have you experienced analytical difficulties due to specific formulations (jelly, soft capsule, liquid formulation, tablet, etc.)? Have you encountered difficulties in other areas (pretreatment, choice of analytical method, use of column, etc.)? What solutions can you suggest for the above-mentioned problems of interactions between composite materials and between raw materials and excipients? What solutions can you suggest for the analytical difficulties due to the formulation characteristics mentioned above? What solutions can you suggest for the above-mentioned preprocessing, analytical method, and column selection? In order to expand health functional food products using functional composite raw materials, what do you think needs to be improved most in the current analytical and quality management system?
Other	Other opinions

**Table 3:** Participant demographics.

Target Group	Division	Category	*N* (%) ^1^
Phase 1: MIDS ^2^ industry workers entry 2	Main task	Analytical	7 (21.2%)
		Quality control	13 (39.4%)
		Research and development	10 (30.3%)
		Materials research	3 (9.1%)
	Experience	<10 years	12 (36.4%)
		10–20 years	9 (27.3%)
		20–30 years	10 (30.3%)
		≥30 years	2 (6.1%)
Phase 2: Academic and industry experts	Main task	Analytical	7 (70%)
		Design and development	3 (30%)
	Experience	<10 years	1 (10%)
		10–20 years	5 (50%)
		20–30 years	3 (30%)
		≥30 years	1 (10%)

^1^ *N* (%) = number of participants (percentage of the total participants in each category) ^2^ MIDS = multi-ingredient dietary supplement

**Table 4:** Summary of the thematic analysis.

Phase	Theme	Sub-Theme	Code
Phase 1: MIDS ^1^ industry workers	Raw material and ingredient	Difficulties in the analysis of raw materials and ingredients	Difficulties in measuring ingredient levels
			Ingredient loss during distribution
			Analytical difficulties due to the presence of isomers
		Analysis and interaction issues in composite ingredients	Content changes due to raw material interactions
			Analytical difficulties due to peak time differences between raw materials
	Formulation	Analytical challenges arising from formulation diversity	Analytical difficulties due to specific formulations
			Ingredient-level changes due to formulation interference
	Testing methods	Limitations of current testing methods	Absence of established test methods
			Lack of raw material-specific guidelines
		Inter-manufacturer differences in chromatography column performance	Column-to-column variation between manufacturers
			Inconsistent column selection criteria
Phase 2: Academic and industry experts	Expert-recommended analytical solutions	Tailored pretreatment and extraction strategies	Solutions to raw material analysis issues
			Solutions to formulation-related challenges
		Regulatory improvements and method development	Improvements in testing methods
			Procedural and institutional improvements

^1^ MIDS = number of participants (percentage of the total = multi-ingredient dietary supplement).

**Table 5:** Summary of the analytical challenges and proposed solutions identified through expert interviews.

Division	Standard Material	Identified Challenges	Proposed Solutions
Vitamin B12	Vitamin B12	Significant reduction or undetectable levels due to accompanying components (Vitamin C, *Rhodiola rosea* L. extracts)	- Adjust sample solution concentration and range - Optimize buffer solvent properties - Verify extraction efficiency across matrices
Vitamin K2	Vitamin K2	Decreased values associated with magnesium as a co-formulant	- Adjust sample solution concentration and range - Optimize buffer solvent properties - Verify extraction efficiency across matrices
Vitamin B9	Vitamin B9	Variations due to differences in standard materials and pretreatment solvents specified in the Standards and Specifications for Health Functional Foods	- Align sample preparation with the standard method - Dissolve in 0.1 N NaOH and titrate with 10 mM phosphate buffer
*Rhodiola rosea* L.	Rosavin	Analytical complexity due to the presence of isomers (rosavin and its isomers)	- Reduce column packing size - Use a buffered mobile phase for better separation
Marigold flower extracts	Lutein, zeaxanthin	Inflated values due to structurally similar carotenoids	- Employ precision analytical equipment - Instead of the total carotenoid analysis (UV–Vis spectrophotometry) and area ratio quantification currently included in the Standards and Specifications for Health Functional Foods, lutein is directly quantified by constructing a calibration curve using lutein standard materials.
*Haematococcus pluvialis* extracts	Astaxanthin	Inflated values due to structurally similar carotenoids	- Employ precision analytical equipment - Instead of the total carotenoid analysis (UV–Vis spectrophotometry) and area ratio quantification currently included in the Standards and Specifications for Health Functional Foods, lutein is directly quantified by constructing a calibration curve using lutein standard materials
Bilberry extracts	Anthocyanosides	Inflated values due to the presence of similar series of accompanying ingredients	- Use precision instrumentation - Separate and exclude the absorbance of interfering components in the final calculation
Cranberry fruit extracts	Proanthocyanidins	Inflated values due to the presence of similar carotenoids	- Use precision instrumentation - Separate and exclude the absorbance of interfering components in the final calculation
Vitamin D	Vitamin D	Interference from multi-ingredient formulations and component non-detection	- Adjust sample solution concentration and range - Optimize buffer solvents - Verify extraction efficiency by matrix
Biotin	Biotin	Interference from multi-ingredient formulations and component non-detection	- Adjust sample solution concentration and range - Optimize buffer solvents - Verify extraction efficiency by matrix
Vitamin C	Vitamin C	Numerical fluctuations caused by the accompanying ingredients	- Adjust sample solution concentration and range - Optimize buffer solvents - Verify extraction efficiency by matrix
Propolis extracts	Total flavonoids	Numerical variations due to interfering excipients	- Adjust sample solution concentration and range - Optimize buffer solvents - Verify matrix-specific extraction efficiency
Saw palmetto fruit extracts	Fatty acid	Numerical fluctuations caused by the accompanying ingredients (e.g., *Ginkgo biloba* leaf extracts)	- Adjust sample solution concentration and range - Optimize buffer solvents - Verify matrix-specific extraction efficiency
Pantothenic acid	Pantothenic acid	Decreased values near the end of shelf life	- Typically consumed within 2 years; degradation not considered problematic
Glutathione	Glutathione	Absence of an official testing method	- Refer to the Korean Pharmacopoeia and international sources (e.g., United States Pharmacopoeia)
Natural ingredients	Natural lemon extract powder	Difficulty in peak alignment between natural samples and synthetic standards	- Minimize impurities in natural ingredients - Identify and address factors influencing retention time
Jelly formulation		Complexity of the extraction process	- Cut into smaller pieces - Adjust the sample amount - Completely dissolve during pretreatment - Account for long-term physical changes - For protein-based gels: disperse in water or buffer, then extract with acetone
Soft capsule		Ingredient migration into capsule shell complicates analysis	- Use dichloromethane or chloroform for separation
Solid formulation		Variation in results due to pretreatment methods	- Use a mortar and pestle to avoid heat from blenders - Homogenize thoroughly before analysis - Remove coating during grinding, if applicable

## Data Availability

The original contributions presented in this study are included in the article/Supplementary Materials. Further inquiries can be directed to the corresponding author.

## Supplementary Materials

The following supporting information can be downloaded at: https://www.mdpi.com/article/10.3390/foods14213598/s1, Table S1. Themes, subthemes and illustrative quotations from the data.

## Abbreviations

The following abbreviations are used in this manuscript:

MIDS/MIDSs	Multi-ingredient dietary supplement(s)
DS/DSs	Dietary supplement(s)
CAGR	Compound annual growth rate
FGI	Focus group interviews
MFDS	Ministry of Food and Drug Safety
N (%)	Number of participants (percentage of the total participants in each category)
SOPs	Standard operating procedures
USP	United States Pharmacopeia
EFSA	European Food Safety Authority
