# Supplementary material for: Expert Perspectives on Enhancing Analytical Methods for Multi-Ingredient Dietary Supplements (MIDS): A Qualitative Study
Source: Foods. 2025 Oct 22;14(21):3598. doi: 10.3390/foods14213598 (PMC12607475; doi:10.3390/foods14213598)
Supplement: Supplementary file 1 [file foods-14-03598-s001.zip › foods-3877894-supplementary.pdf]

Table S1. Themes, subthemes and illustrative quotations from the data

Phase	Theme	Sub-theme	*Illustrative Quotation*
Phase 1: MIDS[1] Industry Workers	Raw Material & Ingredient	Difficulties in the Analysis of Raw materials and Ingredients	*"Trace elements can get buried, and it's very difficult to actually see them." (Professional #1).*<br><br>*"Trace ingredients like vitamin D and vitamin K are sometimes all detected when the content is validated, but in some situations, only half of them are detected." (Professional #7).*<br><br>*"If the overdose is not applied, the content falls below the standard within the expiration date, and stability is compromised." (Professional #1).*<br><br>*"Probiotics were initially allowed, but they are no longer permitted due to side effects." (Professional #4).*<br><br>*"So, they partially accepted raising the upper limit..." (Professional #6).*

		"Please make it higher than the upper limit. If you approve that, we can safely proceed with the analysis..." (Professional #7). <br>*"Vitamin C in Acerola fruit extracts has a different peak time than synthetics, which can cause differences in analysis." (Professional #4).* <br>*"I could see that the test for Rhodiola rosea L. extracts tended to have some impurities in it." (Professional #16).* <br>*"Folic acid, for example, I think it's a little bit affected by pH." (Professional #14).* <br>*"Ginsenosides themselves are affected by acid. It's affected a little bit by temperature and pH, so Rg1 becomes Rg3, and there's damage like that." (Professional #15).*
	Analysis and Interaction	*"When analyzing cranberry fruit extracts, similar anthocyanosides are sometimes detected together in the absorbance, resulting in a high content." (Professional #2).*

	Issues in Composite Ingredients	*"When analyzing lutein, other carotenoids such as astaxanthin, zeaxanthin, and beta-carotene can be detected together, which can skew the results." (Professional #10).*<br><br>*"Lutein checks total carotenoid absorbance using a UV-Vis spectrophotometer. At that time, astaxanthin, zeaxanthin, beta-carotene, and other carotenoids are detected..." (Professional #2).*<br><br>*"Bilberry extracts also have a high content because anthocyanins overlap..." (Professional #1).*<br><br>*"Beta-glucan is a declared form, but because it's mostly present in raw materials, the value doesn't come out right when you formulate with it. Since the beta-glucan analysis method is total glucan minus alpha-glucan, there's only one way to calculate alpha-beta-glucan. If you mix in cellulose or something similar, the value increases..." (Professional #10).*

"When Rhodiola rosea L. extracts and vitamin B12 were prescribed together, the results were not good." (Professional #1).

"When vitamin B12 and vitamin C are prescribed together, the vitamin B12 content is somewhat reduced..." (Professional #2).

"In the case of trace elements such as biotin or vitamin D, the wavelength is low, so they are often buried and interfered with by other elements." (Professional #2).

"If you prescribe vitamin B12 and copper sulfate, the vitamin B12 does not appear." (Professional #7).

"When I prescribe vitamin B12 and milk thistle extracts together, the screen does not appear either." (Professional #7).

	"In the case of saw palmetto and ginkgo leaf extracts, the content does not appear when combined..." (Professional #3).<br><br>*"It's a method that's in the Health Functional Food Code, but when catechins and amino acids were mixed, the process was done according to the process, but when the raw materials were tested alone, the result was 100, but when amino acids were mixed, it didn't even come out half." (Professional #15).*<br><br>*"I once used organic raw materials such as vitamin D and rice bran extract as secondary raw materials, but when the two were mixed together, the vitamin D content did not come out." (Professional #19).*<br><br>*"If you mix vitamin B2 in a high magnesium product, the value of vitamin B2 is a little bit unstable." (Professional #14).*<br><br>*"The flavonoids in propolis extracts are highly interfered with by excipients, and the*

		variations are quite high." (Professional #2).<br><br>*"There was also interference between the individual recognizable forms of Saururus chinensis extract and propolis extracts." (Professional #19).*<br><br>*"I put 100% inputs into the vitamin C product, but there was interference because due to vitamin C in the excipients..." (Professional #3).*<br><br>*"Saw palmetto fruit extracts have a lot of assay variation and doesn't give the right assay." (Professional #11).*<br><br>*"When you mix natural ingredients with natural ingredients, it becomes unstable." (Professional #10).*
Formulation	Analytical Challenges Arising from	*"There are some things that the existing formulations can't cover because the formulations were created first and the jelly formulation was added on." (Professional #2).*

	Formulation Diversity	*"Some of the formulations we've had a little trouble with are jelly." (Professional #14).*<br><br>*"With jelly, you don't melt it all the way through, and then you end up cutting it up, and now you have to analyze it, and depending on how much you cut it up and what size you cut it into, you're going to get different amounts." (Professional #15).*<br><br>*"In the case of jelly, some parts are difficult to dissolve sufficiently. In this case, we are limited to the test methods set for each formulation." (Professional #9).*<br><br>*"We don't make jelly formulations. We have considered the idea, but it is currently on hold. There are technical and content issues..." (Professional #11).*<br><br>*"With water-soluble vitamins in soft dosage forms, there are challenges with water-based pretreatments that do not sufficiently dissolve the ingredients." (Professional #9).*

	"It is not that the ingredient is not actually present, but that it has been transferred to the film. To prove this, you have to repeat experiments for each formulation, but it is difficult to continue this in reality." (Professional #6).<br><br>*"The addition of acid in multivitamin granules or wet formulations can cause dissipation problems, depending on the activity of the water-soluble vitamins." (Professional #4).*<br><br>*"When you dissolve water in a liquid, it turns out well. However, when you create a solid, there are many interfering substances and the water does not dissolve completely, resulting in a deviation." (Professional #4).*<br><br>*"It would be much better if there were such parts. Usually, if you look at the current public notice, the contents of new things are detailed and there are not many problems with new substances, but if you look at the old nutrient standards, there is an update..." (Professional #3).*

		"I also think now that test methods need to be formulation specific." (Professional #13).
Testing Methods	Limitations of Current Testing Methods	*"There is no separate method for glutathione, so it is difficult. Also, all phospholipids are supposed to be checked by the acetone-insoluble method, but when the phospholipids of krill oil are checked by the acetone-insoluble method, the data difference is about 30%, so it cannot be verified." (Professional #1).*<br><br>*"When using the food method and the dietary supplements method, biotin was better analyzed in the food method and so on." (Professional #14).*<br><br>*"The stick jelly melts at 40 to 50 degrees, but that's not in the instructions." (Professional #29).*<br><br>*"I think it would be helpful to have a little more information in the manual about why they used these solvents in the instructions." (Professional #17).*
	Inter-	*"Technical differences between manufacturers can lead to differences in separation,*

		Manufacturer Differences in Chromatography Column Performance	*sharpness, etc., and the retention time itself can vary." (Professional #2).*<br><br>*"There's definitely a difference." (Professional #14).*<br><br>*"The characteristics of the column device are clearly present in the manufacturer's specifications. In the case of individually recognized specified columns, we mainly use the specified columns. Even if the specifications are equivalent to the standard, there are many cases where they do not come out." (Professional #3).*<br><br>*"It is important to stick to the column that was used as a specific reference standard." (Professional #7).*<br><br>*"In theory, it should be fine if the sizes are the same. However, since each company has slightly different column filling technology, there seems to be some discrepancy." (Professional #10).*
Phase 2:	Expert-	Tailored	*"For vitamin B12 analysis, depending on the matrix nature of the sample, an additional*

Academic & Industry Experts	Recommended Analytical Solutions	Pretreatment and Extraction Strategies	*enzymatic hydrolysis treatment is required to free the component from the matrix so that it can be extracted using an enzymatic treatment method such as pepsin... Vitamin K requires different pretreatment or extraction methods, such as lipase enzyme treatment to break down fats present in the sample." (Expert #3).*<br><br>*"The sample pretreatment is the same as for the standard, a certain amount of sample is taken, dissolved with 0.1 N NaOH, and eluted with 10 mM phosphate buffer for analysis... The separation of rosavin isomers and rosavin in Rhodiola rosea extracts is considered possible by reducing the size of the column filler or by using buffer as the mobile phase." (Expert #6).*<br><br>*"In the case of jellies, we are now experimenting with chopping them up as much as possible and controlling the sample volume. In addition, you can try freezing the jelly completely in liquid nitrogen." (Expert #10).*<br><br>*"You can use dichloromethane or chloroform to separate them and then do the*

		experiment." (Expert #7).
	Regulatory Improvements & Method Development	*"The test method generally recommends using general-purpose and universal equipment, but it would be good to consider expensive equipment as well, because there may be expensive equipment or equipment that not everyone can afford." (Expert #5).*<br><br>*"It is good to have multiple analytical methods, but as the number of analytical methods increases, the analyst needs to be responsible for whether it is appropriate to use the method." (Expert #7).*<br><br>*"For the products developed so far, it is necessary to divide them into several types, select a representative matrix, and develop appropriate analytical methods." (Expert #4).*<br><br>*"It will be difficult to apply all methods from the Health Functional Food Code to all matrices, but this part needs to be developed through continuous research as in other countries." (Expert #3).*

[1]MIDS = multi-ingredient dietary supplement
